# Ongoing loss of viable neurons for weeks after mild hypoxia-ischaemia

**DOI:** 10.1093/braincomms/fcaf153

**Published:** 2025-04-18

**Authors:** Melanie A McNally, Lauren A Lau, Simon Granak, David Hike, Xiaochen Liu, Xin Yu, Rachel A Donahue, Lori B Chibnik, John V Ortiz, Alicia Che, Raul Chavez-Valdez, Frances J Northington, Kevin J Staley

**Affiliations:** Department of Neurology, Harvard Medical School and Massachusetts General Hospital, Boston, MA 02114, USA; Department of Neurology, Harvard Medical School and Massachusetts General Hospital, Boston, MA 02114, USA; Department of Neurology, Harvard Medical School and Massachusetts General Hospital, Boston, MA 02114, USA; Department of Radiology, Athinoula A. Martinos Center for Biomedical Imaging, Harvard Medical School and Massachusetts General Hospital, Boston, MA 02129, USA; Department of Radiology, Athinoula A. Martinos Center for Biomedical Imaging, Harvard Medical School and Massachusetts General Hospital, Boston, MA 02129, USA; Department of Radiology, Athinoula A. Martinos Center for Biomedical Imaging, Harvard Medical School and Massachusetts General Hospital, Boston, MA 02129, USA; Department of Medicine, Biostatics, Massachusetts General Hospital, Boston, MA 02114, USA; Department of Neurology, Harvard Medical School and Massachusetts General Hospital, Boston, MA 02114, USA; Department of Epidemiology, Harvard TH Chan School of Public Health, Boston, MA 02115, USA; Department of Psychiatry, Yale University School of Medicine, New Haven, CT 06510, USA; Department of Psychiatry, Yale University School of Medicine, New Haven, CT 06510, USA; Department of Pediatrics, Johns Hopkins School of Medicine, Baltimore, MD 21205, USA; Department of Pediatrics, Johns Hopkins School of Medicine, Baltimore, MD 21205, USA; Department of Neurology, Harvard Medical School and Massachusetts General Hospital, Boston, MA 02114, USA

**Keywords:** neuronal death, mild hypoxic-ischaemic injury, GCaMP, network, neocortex

## Abstract

Mild hypoxic-ischaemic encephalopathy is common in neonates, and there are no evidence-based therapies. By school age, 30–40% of those patients experience adverse neurodevelopmental outcomes. The nature and progression of mild injury is poorly understood. We studied the evolution of mild perinatal brain injury using longitudinal two-photon imaging of transgenic fluorescent calcium-sensitive and insensitive proteins to provide a novel readout of neuronal viability and activity at cellular resolution *in vitro* and *in vivo*. *In vitro*, perinatal organotypic hippocampal cultures underwent 15–20 min of oxygen-glucose deprivation. *In vivo*, mild hypoxia-ischaemia was completed at post-natal day 10 with carotid ligation and 15 min of hypoxia (FiO_2_, 0.08). Consistent with a mild injury, minimal immediate neuronal death was seen *in vitro* or *in vivo,* and there was no volumetric evidence of injury by *ex vivo* MRI 2.5 weeks after injury (*n* = 3 pups/group). However, in both the hippocampus and neocortex, these mild injuries resulted in delayed and progressive neuronal loss by the second week after injury compared to controls; measured by fluorophore quenching (*n* = 6 slices/group *in vitro*, *P* < 0.001; *n* = 8 pups/group *in vivo*, *P* < 0.01). Mild hypoxia-ischaemia transiently suppressed cortical network calcium activity *in vivo* for over 2 h after injury (versus sham, *n* = 13 pups/group; *P* < 0.01). No post-injury seizures were seen. By 24 h, network activity fully recovered, and there was no disruption in the development of normal cortical activity for 11 days (*n* = 8 pups/group). The participation in network activity of individual neurons destined to die *in vivo* was indistinguishable from those that survived up to 4 days post-injury (*n* = 8 pups/group). Despite a lack of significant immediate neuronal death and only transient disruptions of network activity, mild perinatal brain injury resulted in a delayed and progressive increase of neuronal death in the hippocampus and neocortex. Neurons that died late were functioning normally for days after injury, suggesting a new pathophysiology of neuronal death after mild injury. Critically, the neurons destined to die late demonstrated multiple biomarkers of viability long after mild injury, suggesting their later death may be modified with neuroprotective interventions.

## Teaser

Infants with mild hypoxia-ischaemia have significant long-term disabilities, and there are no evidenced-based therapies. Using cutting-edge tools, McNally *et al*. report that mild perinatal hypoxia-ischaemia exacerbates neuronal death for weeks. The neurons that die late after injury show robust viability for many days, indicating an extended therapeutic window.

## Graphical Abstract

**Graphical Abstract:**
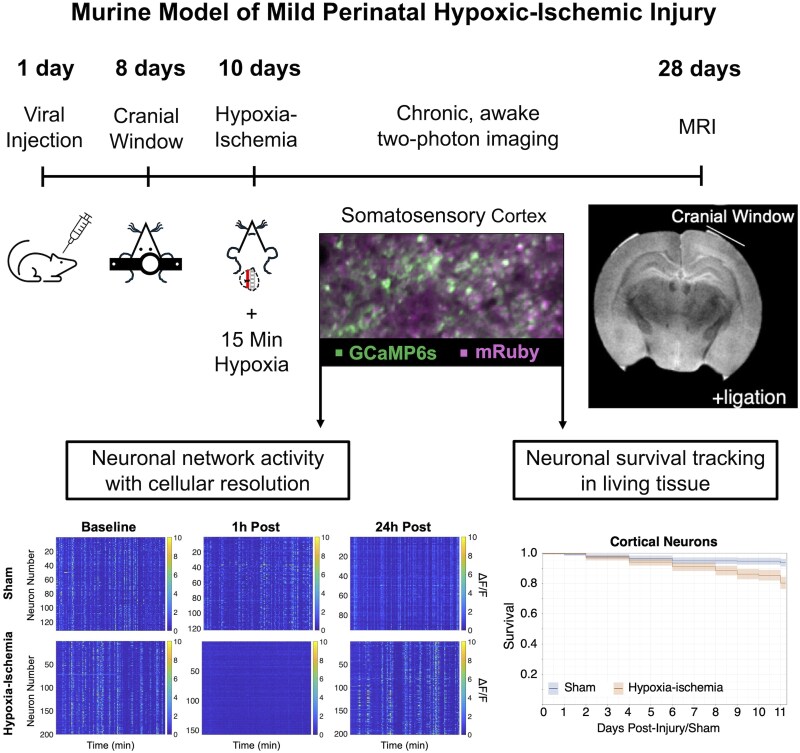

## Introduction

Neonatal encephalopathy due to intrapartum hypoxia-ischaemia is a major cause of neonatal mortality and morbidity with 2–8 in 1000 live births being affected worldwide.^1,2^ Of these infants, most are categorized as having mild encephalopathy, although this incidence is likely underestimated as many mild cases go undiagnosed.^2^ Early studies found that term neonates with mild hypoxic-ischaemic encephalopathy (HIE) had similar long-term neurodevelopmental outcomes to neonates without HIE.^3,4^ However, mild HIE is now diagnosed within the first few hours after birth to determine eligibility for treatment with therapeutic hypothermia, which is reserved for moderate-to-severe encephalopathy.^5,6^ Infants with mild HIE have significant disabilities compared with unaffected peers, with up to 25% having impairments by 18 months and 35–40% by 5 years of age.^7-9^ Thus, the long-term global burden of disability for these patients is significant, and there are currently no therapeutic interventions with demonstrated benefit for these patients. To develop therapies, we first need to understand the progression of injury in these patients.^4,10,11^

Injury progression after *moderate to severe* perinatal hypoxia-ischaemia (HI) has been studied with various modalities in preclinical models and in patients, all having limitations.^12-14^ The relationships between the number of visible dying neurons, the rate of neuronal death, the rate of efferocytosis (i.e. clearance) of dying neurons and the time elapsed since injury are quite complex.^15-18^ Histopathological assays for neuronal death are limited in their ability to achieve the temporal resolution required to quantify this injury and test for progression in real time. Longitudinal studies of neuronal death in living tissue are needed for accurate quantification.^19,20^ Progression of injury is a particularly important question in mild HIE, where the possibility of late progression of neuronal death might alter acute treatment decisions, perhaps opening a longer window of opportunity to intervene.

Injury progression after mild perinatal HI has not yet been elucidated in preclinical studies. Clinically, MRI is used, but it is not sufficiently sensitive to assess for the degree and progression of injury in these mild HIE patients.^7,21,22^ The acute MRI abnormalities detected in ∼20–40% of contemporary mild HIE cohorts are subtle, most often with only small areas of signal change in the white matter or cortex.^21,23,24^ In contrast, acute MRI abnormalities in infants with moderate or severe HIE are more common and more extensive; findings that are predictive of poorer long-term outcomes.^25^ The association of the more subtle injury seen on MRI in infants with mild HIE to childhood outcomes is unknown. EEG has also been used to assess HIE.^26,27^ While its utility studying disease progression after mild HIE is unknown, EEG shows acute abnormalities in up to 70% of infants with mild HIE not undergoing therapeutic hypothermia.^28^ These findings indicate acute dysfunction of injured neural networks. At a cellular level, injured neurons are subject to synaptic stripping^29,30^ and loss of postsynaptic spines,^31,32^ which functionally disconnect the injured neurons from the neural network. The degree of participation of individual neurons in network activity should therefore be a sensitive measure of viability when measuring injury progression longitudinally.

In this study, we used longitudinal assays *in vitro* and *in vivo* to examine sequelae of mild perinatal HI brain injury. Viability of individually tracked neurons was assessed using ongoing expression of transgenic fluorescent proteins *in vivo* and *in vitro*,^15,33,34^ and participation in network activity *in vivo*.^35^ With these tools, we demonstrated a very delayed and progressive neuronal cell loss following mild perinatal HI that is preceded by a period of normal neuronal function, potentially opening a therapeutic window for interventions for mild HIE to prevent life-long adverse outcomes.

## Materials and methods

### Animals

All animal protocols were approved by the Massachusetts General Hospital Institutional Animal Care and Use Committee (protocol #2020N000141). All studies were conducted in accordance with the United States Public Health Service's Policy on Humane Care and Use of Laboratory Animals. Wild-type mice (C57bl/6; Jackson Labs) of either sex were used. Littermates were used, when possible, to minimize potential confounders. On postnatal day 1 (P1), all animals underwent intracerebroventricular injection in the left hemisphere with pAAV1-hSyn1-mRuby2-GSG-P2A-GCaMP6s-WPRE-pA (Addgene, #50942). Mouse pups remained in the home cage with the dam under standard husbandry conditions until P6–8 when organotypic hippocampal slice cultures were prepared. For *in vivo* experiments, pups stayed with their dams under standard husbandry conditions until P7-8 when the cranial window was placed. They were removed from their home cages for subsequent imaging and interventions but returned to their dam between timepoints through P28 at the experimental endpoint.

### *In vitro* organotypic slice cultures, oxygen-glucose deprivation and imaging

Organotypic hippocampal slice cultures were prepared as membrane insert-mounted cultures.^36^ Briefly, hippocampi were obtained from P6-8 mice, cut to 400μm slices, transferred to a covered 6-well dish containing a membrane insert (Millipore-Sigma, #PICMORG50), fed twice weekly (100% media exchange) with neurobasal-A media (Life Technologies Corp, #12349015) supplemented with 500 mM Glutamax (Life Technologies Corp, #35050061), 2% B-27 (Life Technologies Corp, #17504044) and 0.03 mg/mL gentamycin (Life Technologies Corp, #15710072), and incubated at 36.5°C in a 5% CO_2_ incubator with passive humidity control. Cultures were imaged starting at 3–4 days *in vitro* (DIV3-4) within a TC-MIS miniature incubator connected to a temperature controller set to the same atmospheric conditions (Bioscience tools, #TC-1-100-I) and randomly assigned to experimental groups. DIV3-4 was chosen as day 0 for the experiments as this approximates a P10 mouse pup, which most closely approximates the stage of brain maturation of a human term neonate.^37^ For oxygen-glucose deprivation (OGD) at DIV3-4, the media was switched to one without glucose and the chamber was aerated with 95% nitrogen/5% CO_2_ for 15–20 min after a 5 min equilibration period. Longer durations of OGD result in pronounced cell damage and inability to image the slice culture chronically to study injury progression, whereas shorter durations do not produce evidence of injury by caspase activation, neuronal swelling, or cell death.^38^ For caspase experiments, slices were pre-incubated with BioTracker NucView® Blue Caspase-3 Dye for >30 min (Sigma-Aldrich, 5 μL dye: 1 mL media, #SCT102). Two-photon (2P) imaging was performed using a custom-built scanning microscope. 2P images were acquired using custom-designed software (LabVIEW), a scan head from Radiance 2000 MP (Bio-Rad), equipped with a 16X, 0.8NA 3.0WD water-immersion objective (Thorlabs, #N16XLWD-PF), and a mode-locked Ti:Sapphire laser (MaiTai; Spectra-Physics). The objective was mounted to an inverter arm positioned beneath the miniature incubator. During imaging, mRuby was excited at 760 nm, green fluorescent protein was excited at 910 nm and blue caspase dye fluorescence was excited at 820 nm. Emission was detected through three filters: 470/50, 545/30 and 620/100 nm. Images were reconstructed offline in ImageJ (RRID: SCR-003070).

### *In vivo* murine cortical window placement

Using a modified method based on that of Che *et al*.,^39^ cortical windows were placed in P7-8 mouse pups under sterile conditions. Briefly, isoflurane anaesthetized mice were placed on a warmer. The scalp was washed with alternating solutions of 70% ethanol and 10% povidone-iodine. A large section of scalp was removed between the ears to expose both hemispheres and the skull was dried. A custom-made, titanium head plate with an inner diameter of 5 mm was centered over the right somatosensory cortex and adhered with veterinary adhesive (Metabond®, Parkell, #S398, #S396, #S371). A craniotomy was performed with gentle etching using a 0.75 mm lancet (World Precision Instruments, #504072), bleeding was controlled using epinephrine haemostatic pellets (Pascal, #12-100). A 3 mm cranial window (Thomas Scientific, #1217N66) was placed over the exposed cortex and subsequently fixed to the skull first with Vetbond® and then with a second layer of Metabond®. Total anesthesia duration was <60 min per pup. Intra-op and post-op analgesia with carprofen (Patterson, #07-844-7425) was administered.

### Neonatal hypoxic-ischaemic brain injury

In P10 mice, a modified Vannucci model was used to induce HI.^40,41^ P10 was chosen because it most closely mimics the stage of brain maturation of a human term neonate.^37^ Under <10 min of isoflurane anesthesia, unilateral ligation of the right carotid artery was completed followed by a 1-hour recovery period. Pups were subsequently exposed to 15 min of hypoxia at 36°C (FiO_2_, 0.08). Fifteen minutes was the longest duration of hypoxia that reliably permitted chronic imaging of the cortex under the cranial window. Higher rates of mortality and widespread acute cell death manifest by nearly complete loss of neuronal fluorescence emission were a frequent complication after longer periods of hypoxia with this experimental design. Control sham pups underwent the same isoflurane anesthesia exposure, and the right carotid artery was exposed, but not ligated. They underwent the same recovery period and time away from dam. Pups were randomly assigned to experimental groups. In the hands of an experienced practitioner (>2 years), 80–85% survival was achieved through the experimental end point (P28) with an overall 65% success rate (some cranial windows were not of sufficient quality to permit longitudinal imaging). Up to 15% less growth is typical in HI animals compared to sham animals prior to weaning.^40-42^ An exception was one sham animal showed marked weight loss prior to the end of the experiment for unknown reasons and had to be euthanized.

### *In vivo* murine chronic two-photon imaging

Unanesthetized mouse pups were stabilized by attaching the headplate to a fixed fork beneath the objective. During P10-12 recordings, the pups were placed on a warming pad (rectal temps maintained at 36–37°C). At > P14, pups were placed on a rotary treadmill. A custom-made gantry-type 2P microscope equipped with a MaiTai 80 MHz Ti:Sapphire HP 1040S ultrafast laser (Spectraphysics) and a 16X, 0.8NA 3.0WD water-immersion objective (Thorlabs, #N16XLWD-PF) was driven with customized ScanImage software (Vidrio RMR) and used for imaging.^43,44^ High-resolution mRuby z-stacks (10 µm step length) of cortical layers 1–3 were acquired of the somatosensory cortex at each timepoint using excitation at 750 nm. About 1.1 Hz calcium imaging was performed with excitation at 920 nm for 500 frames (7.6 min/timepoint). Emission was detected through 605/70 and 525/50 nm bandpass filters (Thorlabs) for mRuby and GCaMP6s, respectively, before PMT detection (Hamamatsu, #C6438-01).

### Processing and analysis of calcium imaging

Inclusion criteria for calcium imaging included the following: (1) imaging quality sufficient to resolve individual neuronal activity in layer II/III somatosensory cortex and (2) minimum of 24 h of data. Animals were excluded from all analyses if atypical injury was noted (e.g. no imageable tissue under cortical window at P11 or later timepoints, or no neuronal or network activity with sustained high basal calcium signal at P11 or later timepoints). Calcium imaging was processed and analysed using modified methods based on Lau *et al*.^35^ and Che *et al*.^39^ See Supplemental Experimental Procedures for details.

### Fluorophore quenching assay

A representative subset of neurons was selected on baseline imaging for survival tracking. Inclusion criteria for neuronal survival tracking in vitro included the following: (1) imaging quality sufficient to resolve individual mRuby-positive neuronal soma in the pyramidal layer of CA1 on experimental day 0 (DIV3/4) at approximately 50 μm below slice surface and (2) same region well imaged for at least 10 days. Inclusion criteria for neuronal survival tracking *in vivo* included the following: (1) imaging quality sufficient to resolve individual mRuby-positive neuronal soma in layer II/III somatosensory cortex on P10 and (2) same region well imaged through at least P20. Animals were excluded from all analyses if atypical injury was noted (e.g. no imageable tissue under cortical window at P11 or later, or no neuronal or network activity with sustained high basal calcium signal at P11 or later) or if there was spontaneous mortality prior to experimental end point. Every neuron was tracked separately and manually. *In vitro* and *in vivo*, the three-dimensional z-stacks from day 0 (P10) through day 10/11 (P20/21) were aligned for each neuron to account for any XYZ drift and verified using blood vessels and surrounding neurons as fiduciary markers. A neuron was characterized as ‘quenched’ when no fluorescent signal was seen in the location where the neuron was expected (still considered alive if any signal or dim signal remained). Approximately half of the neuronal survival tracking data was blinded, no differences in survival were found between blinded and unblinded datasets.

### Magnetic resonance imaging

At P27-29, experimental mice were deeply anesthetized with inhaled isoflurane in preparation for intracardiac perfusion. They were subsequently euthanized by exsanguination with 0.1 M phosphate buffered solution (PBS) and then perfused with 4% paraformaldehyde (PFA in 0.1 M PBS). Brains were removed and postfixed in the same solution. One to three days prior to imaging, brains were washed in PBS alone. The day of imaging, the brains were transferred to Fluorinert^TM^ FC-40 (Sigma-Aldrich, #F9755) and immobilized in plastic centrifuge tubes. *Ex vivo*  ^1^H MRI of the brains was performed on a horizontal 14T MR scanner (Magnex Sci, UK, 15T 130 AS) located at the Athinoula A. Martinos Center for Biomedical Imaging in Boston, MA, USA. See Supplemental Experimental Procedures for further details on imaging protocols and data processing.

### Immunohistochemistry

The brain samples were post-fixed overnight in 4% PFA followed by cryoprotection in 30% glycerol. Frozen material was serially sectioned on a microtome cryostat (Leica SM2010R) into 100-μm coronal slices, with each stored in a cryoprotective solution (ethylene glycol, glycerol, and disodium phosphate in distilled water) at −20°C until processed. The free-floating sections were rinsed extensively in 0.1 M PBS. Slices were permeabilized with 0.3% Triton-X100 for 1 h, and non-specific immunoreactivity was blocked with 20% bovine serum in 0.1 M PBS for 3 h at room temperature. This was followed by overnight incubation of brain sections with primary antibodies in 0.1 M PBS containing 0.3% Triton-X100, at 4°C. The anti-green fluorescent protein (chicken monoclonal, 1:2000, Abcam #13970) antibody was used as the primary antibody. Sections were subsequently rinsed in 0.1 M PBS and incubated with secondary antibody (anti-chicken AF488, 1:1000, Invitrogen #A11039) in 0.1 M PBS containing 0.3% Triton-X 100 for 2 h at room temperature. After exposure to secondary antibody, sections were rinsed in 0.1 M PBS and cover slipped in DAPI Fluoromount-G® (Southern Biotech, #0100-20).

### Cortical neuron co-expression of GCaMP and mRuby

To assess co-expression of GCaMP and mRuby in cortical neurons, GCaMP expression was visualized via immunohistochemistry with anti-GFP, while mRuby's intrinsic fluorescent properties were utilized for detection. Images were acquired using Olympus FV3000 confocal microscope with a 60× objective. In a single experimental animal, six distinct cortical regions of interest in layers II/III measuring 212 μm × 212 μm containing GCaMP and mRuby-positive neurons were selected and analysed in ImageJ.

### Statistical analysis

Statistics were performed using Prism 10 software (Graphpad) and R, and graphs were generated in Prism 10, R, and MATLAB. For all analyses, *n* refers to the number of animals, hippocampal slices or neurons as is specified throughout. Normality tests (Shapiro-Wilk test) were first performed. For data sets that follow normal distribution, statistical significance was determined using either Student’s unpaired *t*-tests (indicated on graphs with asterisks, values defined in legends and results) or ANOVA (indicated by *P* values in legends as well as results). For multiple comparisons tests following ANOVA, multiplicity adjusted *P* values were reported (indicated on graphs with asterisks and defined in legends). Means and standard deviations were reported for all results unless otherwise specified. Survival analyses were completed using the Wald test to determine the hazard ratios, *P*-values and z-scores as defined in the legends and results. Due to some animals being used for both control and OGD *in vitro* and animals from the same litter being used for sham and HI, to control for within-animal effect and avoid pseudoreplication of the data, a cox proportional hazard model was performed with clustering on mouse identifier for survival analyses. Similarly, for analysis of neuronal network participation, a linear mixed effect (LME) model was used with each pup included as a random effect, with neurons nested within each pup to control for animal-specific differences.

## Results

### Injury severity optimization

**Figure 1:**
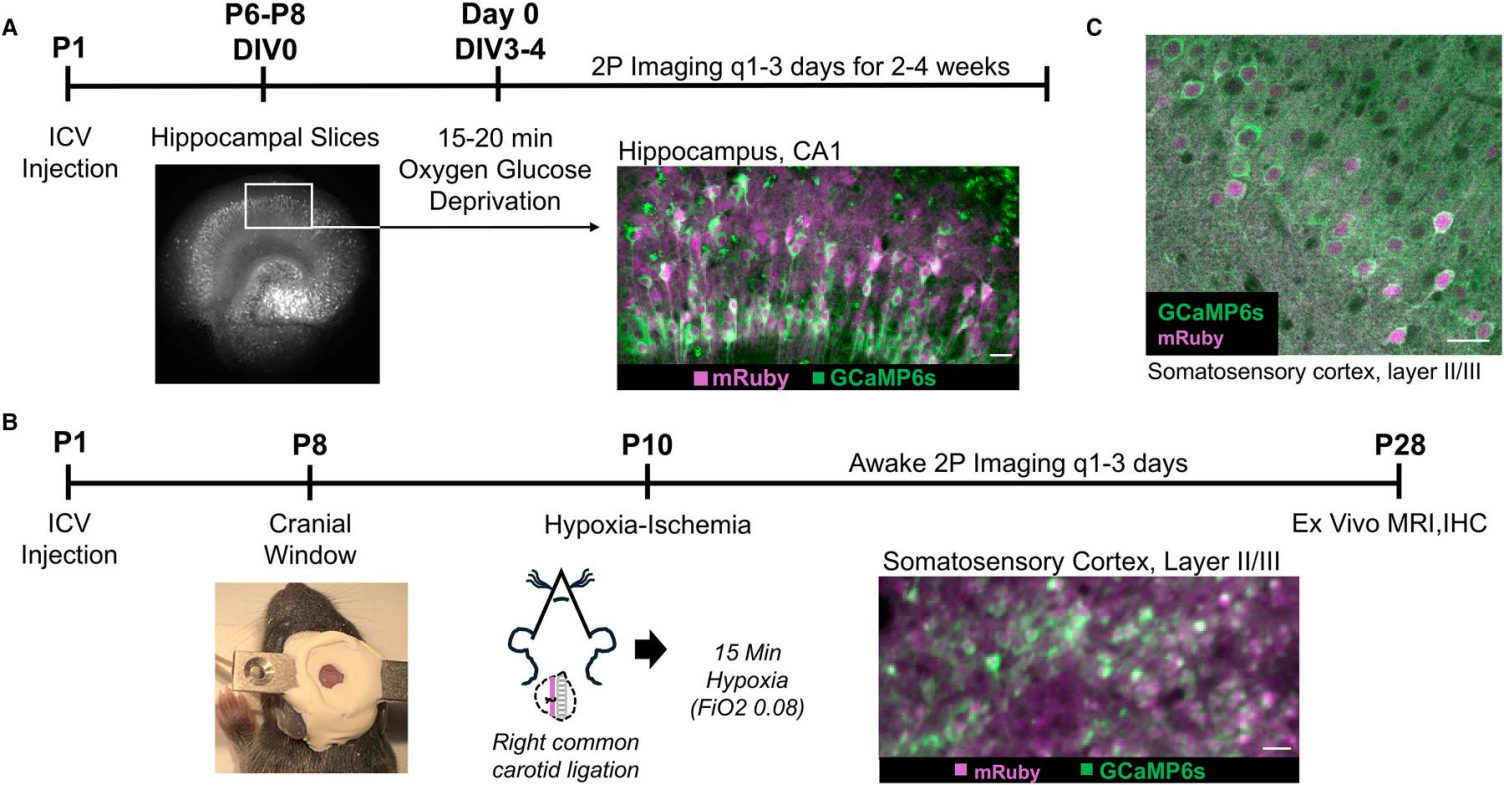
***In vivo* and *in vitro* experimental designs.** (**A**) *In vitro* experimental design with representative hippocampal two-photon (2P) fluorescence images of *syn*-GCaMP6s expression (standard deviation projection of GCaMP6 s activity; white and green) and *syn*-mRuby (magenta). Scale bar on merged images = 25 μm. Intracerebroventricular injection (ICV), days *in vitro* (DIV), post-natal day (P), **m**agnetic resonance imaging (MRI), immunohistochemistry (IHC). (**B**) *In vivo* experimental design with image of mouse pup with implanted custom designed titanium head bar and cranial window. Additional representative layer II/III somatosensory cortex 2P fluorescence image of merged *syn*-GCaMP6s standard deviation projection (green) and *syn*-mRuby (magenta) is shown. Scale bar = 25 μm. (**C**) Representative immunostaining confocal image of *syn*-driven neuronal GCaMP6 s (detected by anti-GFP) and mRuby co-expression in layer II/III somatosensory cortex of a P25 animal post-hypoxia-ischaemia (HI). Manual quantification verified 74% co-expression (*n* = 136 neurons). Scale bar = 25 μm.

Given the inherent challenges of chronic hippocampal 2P imaging *in vivo* in an injured developing mouse pup, an *in vitro* preparation was utilized to track survival of hippocampal neurons after a mild insult. *In vitro*, previously published work from our laboratory tested multiple durations of OGD to identify a mild insult that triggered minor immediate neuronal death, manifest as quenching of fluorescence emission. This level of injury enabled longitudinal imaging of delayed neuronal death by tracking fluorescence emission in the surviving neurons.^38^ With OGD durations ≥25 min, organotypic hippocampal slice cultures exhibited significant cell damage and early quenching. Neurons lost fluorescence emission after 2–3 h post-injury, prohibiting the study of chronic injury progression, whereas 10 min of OGD did not show evidence of any injury (no swelling, dying neurons or caspase activation).^38^ Fifteen to 20 min of OGD was found to cause a small amount of caspase activation and neuronal swelling, but most neurons survived and continued to express fluorescent proteins.^38^ Thus, this duration was selected for the experiments in this study to measure chronic injury after a mild insult *in vitro* (Fig. 1A). *In vivo*, a similar duration of hypoxia (15 min) after carotid ligation permitted longitudinal assessment of cortical neuron injury in the somatosensory cortex (Fig. 1B and C). Longer durations of hypoxia resulted in greater mortality and more extensive acute tissue injury under the cranial window, prohibiting longitudinal imaging and quantification of injury progression using individual neuronal quenching with cellular resolution.

### Neuronal death after mild HI was delayed until second week after injury

**Figure 2:**
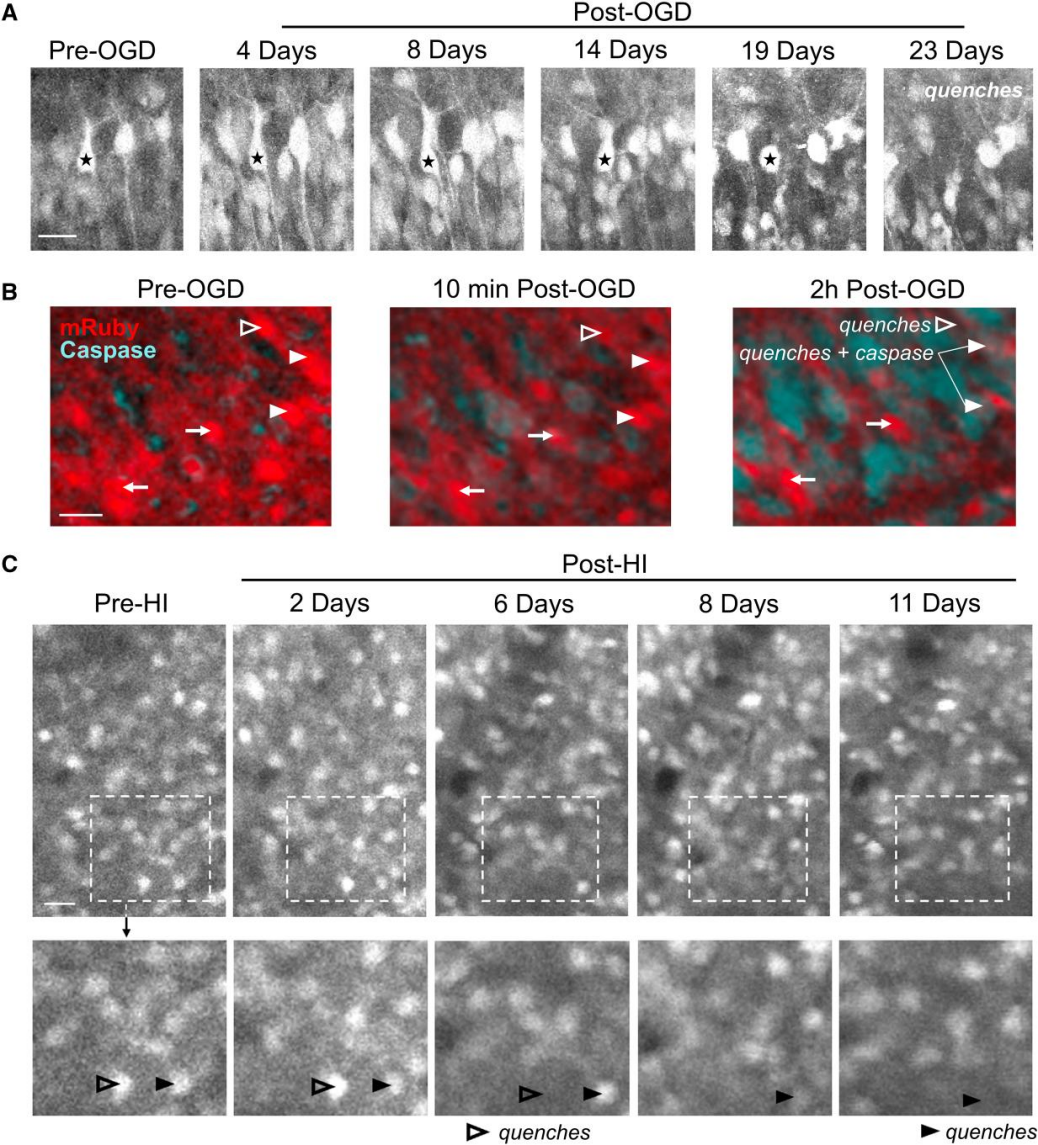
**Longitudinal tracking of hippocampal and cortical neuron survival.** (**A**) Two-photon (2P) fluorescence images tracking mRuby-positive hippocampal pyramidal neurons *in vitro* after oxygen-glucose deprivation (OGD). The starred neuron showed dendritic retraction and swelling prior to quenching at 23 days. (**B**) Representative 2P fluorescence images of mRuby-positive hippocampal pyramidal neurons *in vitro* incubated with NucView® Blue Caspase-3 dye. Neurons that survived post-OGD (arrows) did not show caspase activation acutely. Neurons are shown that quenched 2 h post-OGD with (filled arrowhead) and without (open arrowhead) caspase activation. (**C**) Unanesthetized, *in vivo* 2P fluorescence images of layer II/III somatosensory cortex over time after hypoxia-ischaemia (HI) demonstrating tracking of mRuby-positive cortical neurons. Shown are examples of neurons that quenched 6 days post-HI (open arrowhead) and 11 days post-HI (closed arrowhead). Scale bars = 25 μm.

**Figure 3:**
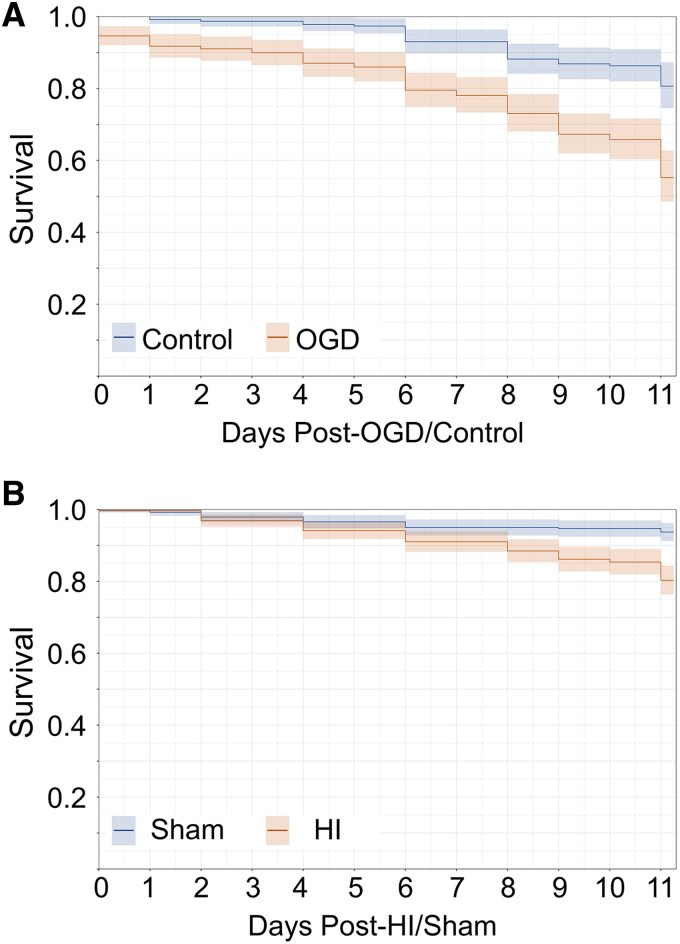
**Persistently elevated rate of hippocampal and cortical neuronal death after mild perinatal hypoxia-ischaemia.** (**A**) *In vitro* hippocampal neuron Kaplan-Meier curve for control conditions (*n* = 6 slices, 227 neurons, 26–49 neurons/slice) versus oxygen glucose deprivation (OGD) (*n* = 6 slices, 277 neurons, 42–56 neurons/slice). In a model controlling for within-animal variation, the risk of neuronal death was significantly higher after OGD compared to control conditions [*P* < 0.001, hazard ratio 2.88 (95% CI = 1.6, 5.19), *z* = 5.4, Wald test]. (**B**) *In vivo* cortical neuron Kaplan-Meier curve for sham [*n* = 8 pups (5 female, 3 male), 376 neurons, 21–79 neurons/pup] versus hypoxia-ischaemia (HI) [*n* = 8 pups (3 female, 5 male), 389 neurons, 18–85 neurons/pup]. In a model controlling for within-animal variation, the risk of neuronal death was significantly higher after mild HI compared to sham [*P* < 0.001, hazard ratio 3.242 (95% CI = 1.8, 5.84), *z* = 4.9, Wald test].

Ongoing emission of transgenically expressed fluorescent proteins has been shown to be a reliable biomarker of neuronal health in vitro,^15,34^ and can be tracked by longitudinal 2P imaging *in vivo* in the developing mouse cortex to identify neurons which undergo programmed neuronal death.^45^ Thus, using chronic 2P imaging of neurons expressing the constitutively fluorescent mRuby, hippocampal and cortical neuronal survival was manually tracked across z-planes in the same field of view using the fluorophore quenching assay after OGD *in vitro* (Fig. 2A and B) and after HI *in vivo* (Fig. 2C and Supplementary Fig. 1). Hippocampal pyramidal neurons that showed robust fluorophore expression after injury *in vitro* did not activate caspase activity acutely (Fig. 2B). When a neuron quenched *in vitro*, the caspase dye activated in the cytoplasm and then translocated to the nucleus where nuclear fragmentation was seen at 24 h consistent with apoptotic neuronal death (Supplementary Fig. 2). Controlling for within-animal effect across experiments, the hazard ratio of death *in vitro* was 2.88 (95% CI = 1.6, 5.19, *z* = 5.4, *P* < 0.001, Wald test) after OGD and *in vivo* was 3.232 (95% CI = 1.8, 5.84, *z* = 4.9, *P* < 0.001, Wald test) after HI (Fig. 3A and B). In both *in vivo* and *in vitro* experiments, the Kaplan-Meier curve shows that the progressively and persistently elevated neuronal death post-injury was most pronounced after the first week (Fig. 3A and B).

### No moderate or severe injury seen on *ex vivo* magnetic resonance imaging

**Figure 4:**
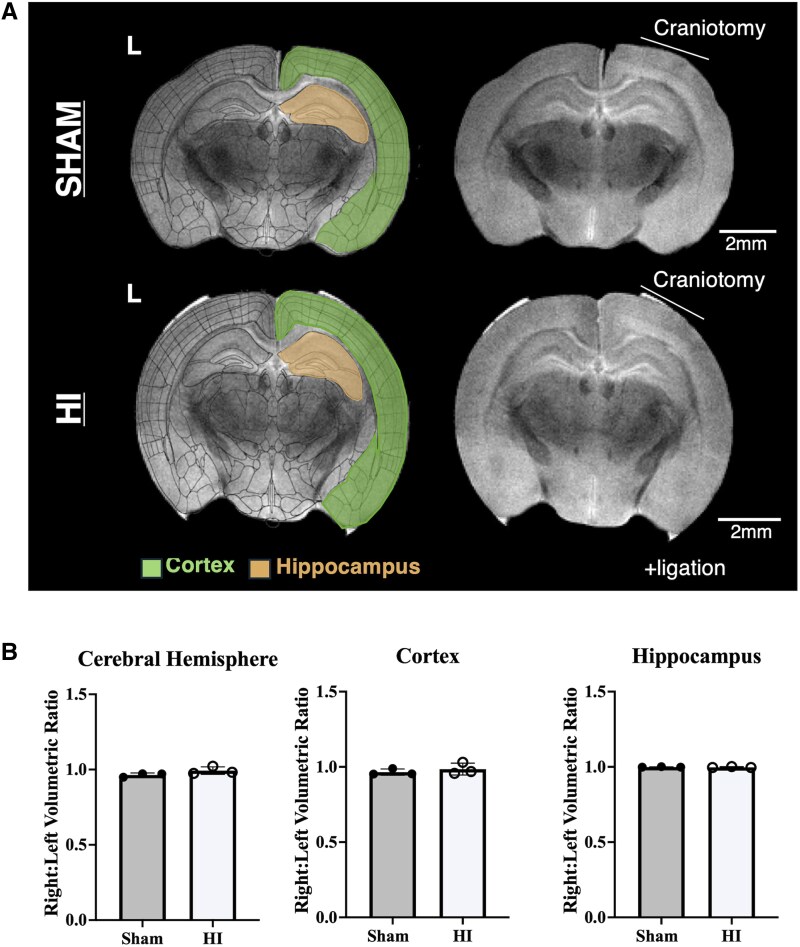
**No moderate or severe injury detected by *ex vivo* MRI after mild perinatal hypoxic-ischaemic injury.** (**A**) Representative T2-weighted magnetic resonance imaging (MRI) coronal slices from sham and hypoxia-ischaemia (HI) animals at location of two-photon imaging. Overlays of the measured regions of interest for volumetric measures at that slice are shown. (**B**) No asymmetries were seen between cerebral hemispheres (*t* = 1.7, df = 4, *P* > 0.05, unpaired *t*-test), cortices (*t* = 0.78, df = 4, *P* > 0.05, unpaired *t*-test) or hippocampi (*t* = 0.63, df = 4, *P* > 0.05, unpaired *t*-test) in sham (*n* = 3, all female) or HI animals (*n* = 3, 2 female and 1 male) 18 days post-injury (P28; mean ± standard deviation). Each data point represents the respective values from a single animal.

*In vivo*, in animals that survived 2 weeks post-HI or sham, no significant visible right hemispheric hypoplasia was seen by gross inspection [*n* = 8 HI (3 female, 5 male), 8 Sham (5 female, 3 male)]. To confirm the absence of moderate to severe injury and validate this observation, *ex vivo* 14 tesla (T), T2-weighted MRI in 3 HI (2 female, 1 male) and 3 sham animals (3 females) 18 days post-injury (P28) (Fig. 4A). There were no differences in the cortical, hippocampal or cerebrum volumes (Supplementary Table 1) and no volumetric asymmetries between the ipsilateral (right) and contralateral (left) cerebral hemispheres (*t* = 1.7, df = 4, *P* > 0.05, unpaired *t*-test), cortices (*t* = 0.78, df = 4, *P* > 0.05, unpaired *t*-test) or hippocampi (*t* = 0.63, df = 4, *P* > 0.05) in either sham or HI animals (Fig. 4B). In contrast, previously published cerebral and regional volumes measured with MRI after moderate to severe HI show significantly decreased volumes in the ipsilateral hemisphere compared to sham animals as early as 8 days post-injury (P18).^41^ Our findings are consistent with (1) clinical data from patients with mild HIE, where MRI abnormalities, when present, are most often subtle^21,24^ and (2) prior *in vivo* MRI studies in both preterm and in utero rodent models of mild perinatal insults showing no macroscopic injury 1–3 months after injury.^46,47^

### Cortical network activity was transiently disrupted after mild hypoxic-ischaemic injury

**Figure 5:**
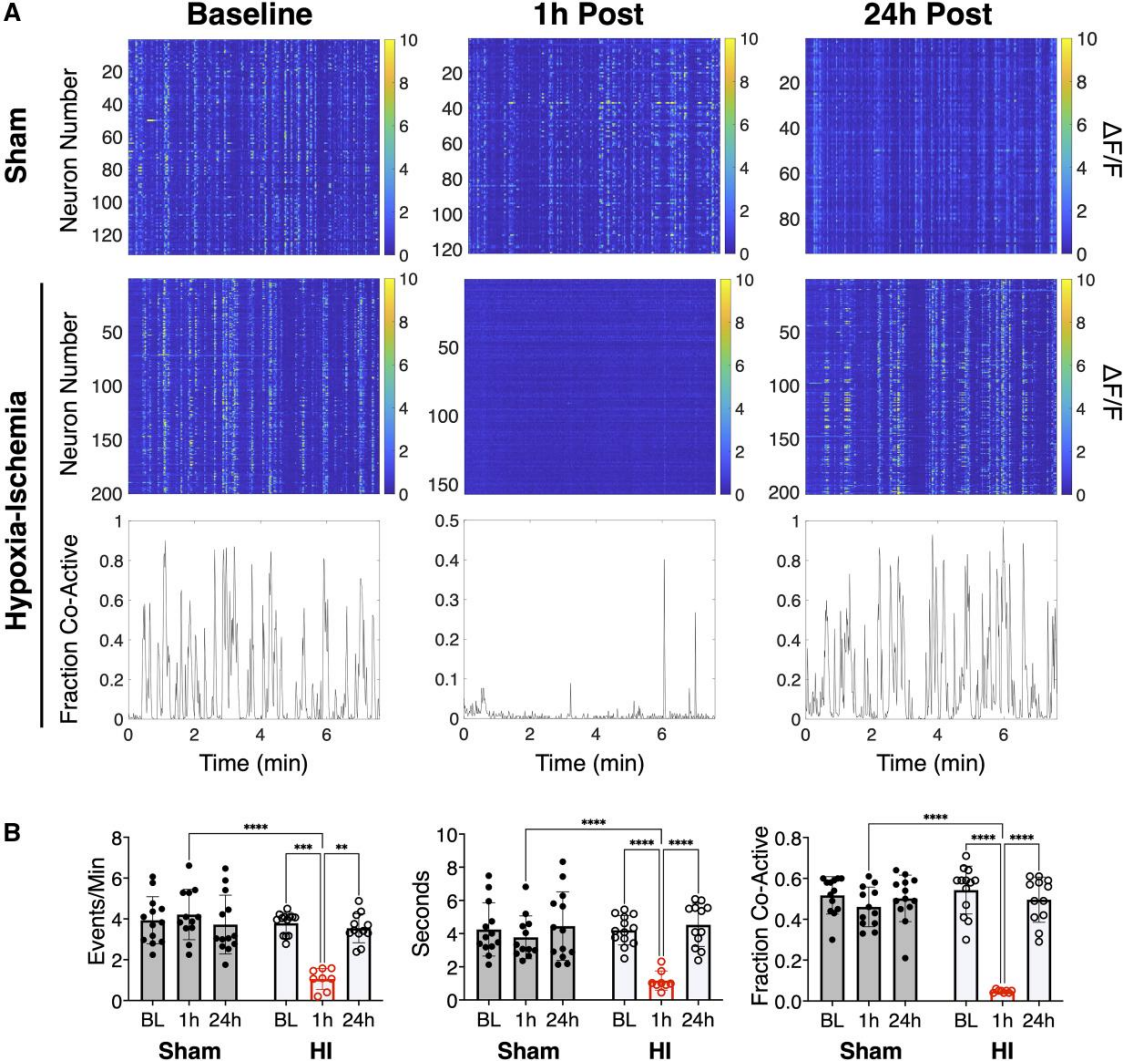
**Mild perinatal hypoxia-ischaemia transiently disrupted cortical network activity.** (**A**) Representative raster plots of ΔF/F *syn*-GCaMP6 s calcium signal taken at 1.1 Hz from one sham and one hypoxia-ischaemia (HI) animal at baseline, 1 h post, and 24 h post timepoints. Representative, corresponding traces to HI raster plots of the fraction of co-active neurons at baseline, 1 h-, and 24 h-post HI are also shown. (**B**) Mean ± standard deviation of network calcium activity metrics in HI [*n* = 13 (6 male, 7 female), open circles] versus sham [*n* = 13 (7 male, 6 female), filled circles] animals at baseline (BL), 1 and 24 h timepoints. The frequency and duration of network events per animal is shown. Network activation is the average proportion of co-active cells per network event per animal. Two-way ANOVA [network event frequency *F*(2, 41) = 21.3, *P* < 0.01; network event duration *F*(2, 41) = 9.9, *P* < 0.001; network activation *F*(2, 41) = 47.4, *P* < 0.01] with *post hoc* Tukey tests, ****P < 0.0001, *** *P* < 0.0001, ** *P* < 0.002.

In human neonates, early cortical network activity is highly synchronous as seen in the well-known developmental EEG patterns of ‘trace discontinu’ and ‘trace alternant’.^48^ Like humans, early murine cortical networks are highly synchronous with frequent spontaneous and responsive correlated network activity critical for normal maturation of cortical circuits.^49,50^ To measure the effects of mild HI on this cortical network activity in vivo, we performed unanesthetized head-fixed 2P neuronal GCaMP6 s calcium imaging of layer II/III in the right somatosensory cortex through a cranial window. Consistent with clinical studies showing that up to 70% of neonates with mild HIE have abnormal EEG findings,^28^ network activity was suppressed and decorrelated acutely and then recovered by 24 h post-HI [*n* = 13 HI (7 female, 6 male), 13 Sham (7 male, 6 female), Fig. 5A and B].

At baseline, synchronous cortical network events were detected at a rate of 3.9 ± 1.2 events/minute in shams and 3.8 ± 0.5 events/minute after HI (Fig. 5B). The average duration of these events at baseline was 4.3 ± 1.6 s in shams and 4.2 ± 0.9 s after HI. The average proportion of co-active cells per network event, ‘Network Activation’, was 0.52 ± 0.09 in shams and 0.54 ± 0.12 after HI. One hour after HI, the frequency of, duration, and neuronal participation in network events decreased compared to baseline and sham, followed by full recovery at 24 h after HI. No seizures were observed during the 2-hour imaging period after HI or during any later timepoints.

### Mild perinatal hypoxic-ischaemic injury did not interfere with maturation of normal cortical and hippocampal activity

**Figure 6:**
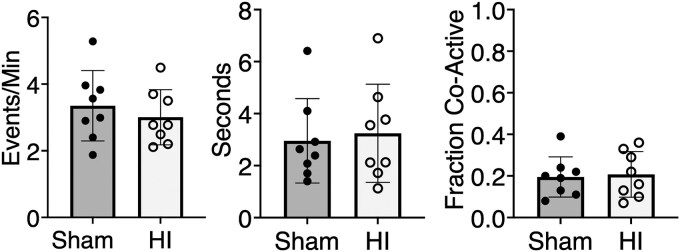
***In vivo* synchronous cortical activity was preserved 11 days after mild perinatal hypoxic-ischaemic injury.** Mean ± standard deviation of network calcium activity metrics in hypoxia-ischaemia (HI) [*n* = 8 (3 female, 5 male), open circles) versus sham (*n* = 8 (5 female, 3 male), filled circles] animals 11 days after injury. The frequency and duration of cortical events per animal is shown. Cortical activation represents the average proportion of co-active cells (‘Fraction Co-Active’) per network event per animal. No differences were detected between groups for cortical event frequency (*t* = 0.73, df = 14, *P* = 0.47, unpaired *t*-test), cortical event duration (*t* = 0.33, df = 14, *P* = 0.75, unpaired *t*-test) and cortical activation (*t* = 0.24, df = 14, *P* = 0.81, unpaired *t*-test).

Longitudinal 2P calcium imaging *in vivo* showed that mild HI did not prevent maturation of normal cortical activity and functional assembly patterns 11 days after injury (Fig. 6). At baseline (P10) through 4 days (P14), the synchronous cortical activity shown in Fig. 5 includes intrinsically generated network events which coexist with those triggered by sensory experience (including passive whisker deflections and spontaneous myoclonic twitches).^49,51^ By 11 days after injury (P21), cortical networks have decorrelated and the synchronous activity measured primarily represents functional neuronal assemblies that are triggered by receptive input such as active whisking and movement on the treadmill while the head-fixed animal was being imaged.^52^ The frequency, duration and proportional co-activation of synchronous triggered cortical events were not different between sham and HI animals *in vivo* 11 days after injury (Fig. 6). Similarly, in a small subset of *in vitro* hippocampal slices, mild OGD did not appear to affect synchronous hippocampal network event frequency, duration, or proportional co-activation 11–12 days after injury *in vitro* compared to control conditions (Supplementary Fig. 3).

### Cortical neurons destined to die participated in physiological network activity for extended periods after injury

**Figure 7:**
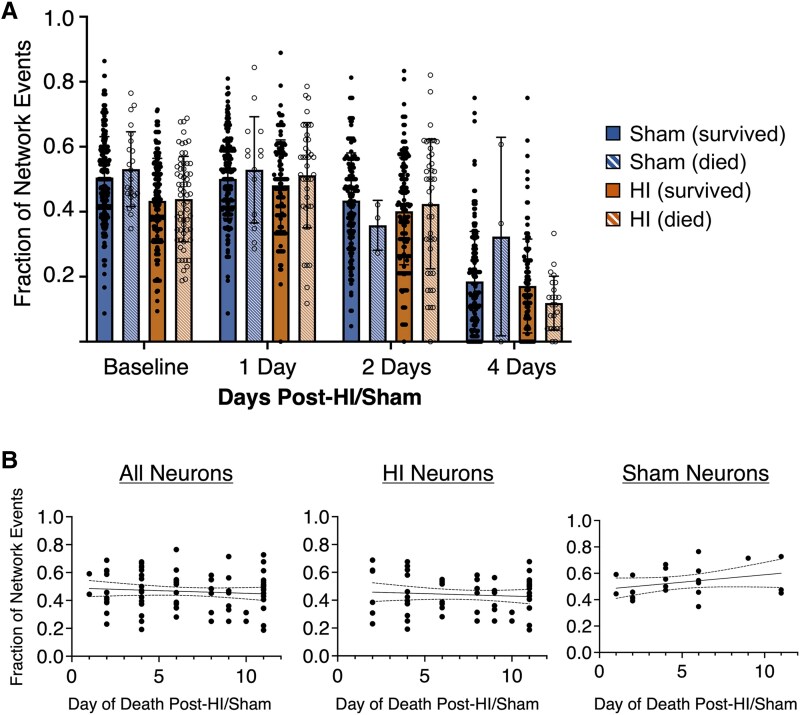
**No difference in network participation of cortical neurons that die versus survive after mild perinatal hypoxia-ischaemia *in vivo*.** (**A**) The fraction of total network events that *individual* neurons were active during (‘neuronal network participation’) was plotted for neurons that quenched prior to day 11 (‘died’, open circles) post-sham (blue striped) or post-hypoxia-ischaemia (HI, orange striped) versus those neurons that survive past day 11 (‘survived’, filled circles) post-sham (blue) or post-HI (orange). Data are shown as mean ± standard deviation at each timepoint after mild HI injury or sham for each group (*n* = 6–8 pups/group/timepoint, 3–333 neurons/group/timepoint). Neuronal network participation of *individual* neurons destined to die was not different from neurons that survived in either sham or HI animals overall, or at baseline, 1-, 2-, and 4-days post-HI (HI, *F* = 0.29, df = (1,16), *P* = 0.60; Sham *F* = 1.72, df = (1,14), *P* = 0.21; Type III F-test for fixed effect, linear mixed effects (LME) models used to account for correlation within pups]. Each data point represents the value from a single neuron. (**B**) Baseline neuronal network participation was not predictive of later neuronal death. Associations between neuronal network participation at baseline (P10) versus day of death are shown for all neurons. LME models, accounting for correlation within pups, showed no statistical associations among all neurons [16 pups, 85 neurons; *F*-statistic = 0.74, df (1,69), *P* = 0.39], HI neurons [8 pups, 62 neurons; *F*-statistic = 0.43, df = (1,53) = *P* = 0.51] and sham neurons [8 pups, 23 neurons; *F*-statistic = 2.25, df = (1,15), *P* = 0.15]. Ninety-fifth percentile confidence limits shown with dashed lines. Each data point represents the value from a single neuron.

We hypothesized that cortical neurons destined to die 1–11 days after HI might undergo early synapse dysfunction and/or dendrite retraction leading up to their commitment to programmed cell death,^53,54^ thus interfering with their participation in synchronous network activity. To test this hypothesis in vivo, we measured the fraction of network events that each manually tracked neuron participated in (‘Neuronal Network Participation’) over time. The analyses were completed *by neuron*, using an LME model controlling for correlation within pups to measure the effects of survival status and treatment group on neuronal network participation. When looking at sham and HI neurons separately, neuronal network participation of *individual* neurons destined to die was not different from neurons that survived in either group overall or at baseline, 1-, 2-, and 4-days post-HI [Fig. 7A; HI Type III test of fixed effect: *F* = 0.29, df = (1,16), *P* = 0.60; Sham *F* = 1.72, df = (1,14), *P* = 0.21; Type III *F*-test for fixed effect]. In neurons with ongoing fluorescent protein expression that died at later times after HI, their network activity participation was unaffected up to 4 days after mild injury. At 4 days (P14), the declining fractions of events in which neurons participate in after mild HI or sham reflects physiological desynchronization.^51,55^ Notably, mild HI did not disrupt this maturational process.

Overall, we did find a statistically significant difference in neuronal network participation between sham and HI neurons [*F* = 23.32, df = (1, 31), *P* < 0.01; Type III test of fixed effect *F*-test]; however, this was primarily driven by differences between the groups at baseline (*t*-statistic = −0.39, df = 49, Tukey adjusted *P* < 0.01; difference of least squared means test). Since baseline differences may have been a confounding variable on neuronal survivability after HI or sham procedures, we completed an LME analysis, accounting for correlation within pups, on neuronal network participation using time as a predictor among the sham and HI neurons. No relationship between neuronal network participation at baseline and time of neuronal cell death was detected (Fig. 7B). No differences in sham versus HI groups were found at later timepoints (*P* > 0.05, LME model).

## Discussion

In the present study, we showed that a mild perinatal HI injury, defined experimentally by the lack of widespread immediate neuronal death, caused a late and persistent elevation of hippocampal and cortical neuronal death through at least the second week after injury despite only transient disruption of cortical network activity and no significant injury on MRI. Here, we show for the first time full recovery of cortical network activity by 24 h after mild HI in vivo. Mild HI did not affect maturational cortical network desynchronization or later cortical responses to sensory input. Additionally, mild HI did not result in frank volume loss on MRI 18 days after injury. Lastly, hippocampal and cortical neurons destined to die after mild HI showed markers of viability for up to 10 days after injury including robust fluorophore expression and full participation in network activity.

Early studies examining the impact of *mild* HI (5 min of ischaemia) on cortical and hippocampal neuronal activity in adult gerbils show 15–30 min of suppression followed by recovery with ongoing normal EEG activity detected as late as 48 h after ischaemia.^56^ Contemporary studies of cortical EEG before, during, and for 2 h after *moderate to severe* perinatal HI (carotid ligation and 45 min of hypoxia) in neonatal mice show decreased EEG background amplitudes compared to baseline for 2 h after injury.^57^ Notably, ∼50% of these animals experience electroconvulsive seizures after moderate to severe HI. Suppression of network activity in the 2 h after mild HI with subsequent recovery by 24 h (Fig. 3) is consistent with both this early and contemporary work.^56,57^ We validated these findings with cellular resolution imaging of cortical networks to measure the recovery of this activity after mild HI for the first time. No acute seizures were seen in the hours after injury. This lack of seizures is a critical defining feature of human mild HIE.

The possibility that neurons survive initial insults only to succumb later has been of great interest because it suggests that there is an extended window of therapeutic opportunity for neuroprotective interventions. In early studies, ‘delayed’ neuronal death after ischaemia in rats and gerbils is seen maximally 48–72 h after injury in hippocampal and cortical neurons.^58,59^ At a gross level in a perinatal injury model employing prolonged (severe) hypoxia and ischaemia, there is ongoing, progressive cerebral volume loss for months, raising the possibility of ongoing neuronal death after injury.^13^ However, efferocytosis of dying neurons and scar formation and retraction likely also contribute to these changes. Subsequent studies have demonstrated dying neurons histopathologically for up to 7 days after moderate to severe HI.^12,14,60^ However, these single time-point histological assays cannot determine the precise time point at which neurons fully commit to cell death and are beyond rescue.^15,18^ Ours is the first definitive demonstration of ongoing, new irreversible commitment to programmed neuronal death up to 11 days after mild HI prior to the actual death of the neuron. The lack of volumetric changes on MRI 18 days after injury confirmed that this was a mild injury, as mice exposed to longer periods of hypoxia have well-established volume changes in the ipsilateral hemisphere detected by MRI by this age.^41^ Additionally, the absence of macroscopic abnormalities at the timepoint studied does not rule out loss of tissue volumes at later times. It is remarkable that the fluorophore quenching assay was sensitive enough to detect the mild cortical injury at this time. Longer-term MRI studies are needed to determine the impact of mild HI on brain development and organization, including functional connectivity. Persistent elevation of neuronal death for weeks or months after injury could lead to increased network simplification, which may be pathological and contribute to the long-term cognitive deficits seen at later ages in these patients.

Studying neuronal death after injury using histopathological assays is complex given dynamic changes in the number of neurons that are ‘visibly dying’ on these assays, the rate of commitment to programmed cell death prior to the assay, the rate of efferocytosis of dying neurons and the time elapsed since injury.^15^ Tracking individual neurons in real time over days and weeks allowed us to overcome a number of these limitations in studying neuronal death.^33^ Progressive injury has been hypothesized for decades after perinatal HI, and this progression forms the theoretical basis for the evolving depth of encephalopathy seen clinically over the first week after injury in both perinatal^4^ and mature^61^ brains. The current study evaluated only mild HI injury, but it was the first to definitively show that progression on a cellular level with a prolonged time window. Perhaps the most important new finding is that the neurons that died late after HI showed evidence of intact function and strong viability on both the single cell and network level. These neurons participated in physiological network activity and demonstrated normal maturation for at least 4 days after injury. These neurons demonstrated functional translational machinery to produce transgenic proteins, absence of caspase activation acutely, and functional connections to the cortical network indicating robust ongoing synaptic activity. In total, the findings suggest an extended therapeutic window for mild HI,^6^ as these neurons were likely salvageable for days after injury if a therapeutic intervention had been available.

The cellular mechanisms of late neuronal death after mild HI remain to be discovered. Developmental neuronal apoptosis in both the neocortex and hippocampus is largely complete at the time points studied,^62-65^ although a recrudescence or exacerbation of these processes is a possible driver of late neuronal death after mild HI in the neonate. It is also possible that mild HI exacerbated isoflurane related neurotoxicity in our animals. Prior studies of P7 mouse pups exposed to 5 h of isoflurane demonstrated significant apoptotic neuronal death in cortical neurons immediately after exposure.^66^ Our HI and sham pups were equally exposed to <1 h of isoflurane at that age for cranial window placement, and then another 5–10 min at P10 for either ligation or sham procedures. This is a lower overall exposure to isoflurane than used by Istaphanous *et al*.,^66^ but this remains a possible confounding variable to explain neuronal death in the control animals. Another consideration is that our cellular-level recordings of network activity did not suggest an activity-dependent mechanism of cell death.^45,67^ Additionally, it is also possible that neurons are not the primary injury site, but rather HI-mediated injury and dysfunction to oligodendroglia or other immune-mediated dysfunction may have caused the secondary loss of neurons that we measured. The mechanisms of programmed neuronal cell death in the developing brain are diverse and the subject of intense study.^14,68,69^ Future studies will need to determine how crucial these various programs are to late neuronal death after mild HI. Understanding these mechanisms is critical for evaluation of therapeutic options to minimize late neuronal death after mild HI.

Our observations regarding late neuronal death were subject to practical limitations of the duration and locations which neuronal death and activity could be tracked in the rapidly developing brain. We did not detect any detrimental effects of the persistent elevation of neuronal death on cortical activity 11 days after injury, or macroscopically by MRI at 18 days after injury, which might explain the later neurodevelopmental deficits seen in human neonates after mild HIE.^7^ It is possible that the metrics of network activity we employed may have missed important developmental transitions that will require more detailed analyses. Our normal MRI findings post-HI are consistent with human studies of neonates with mild HIE, where the majority of MRIs show no abnormalities.^21,23,24^ However, future studies looking at diffuse tensor imaging and tractography may uncover more subtle white or grey matter injury our sequences were not sensitive enough to detect. Additionally, a limitation of the present study is the lack of long-term behavioral outcomes. Defining these outcomes is important to assess the clinical translatability of our findings. Future studies can test whether neurobehavioral deficits are present in animals subjected to the injury severity that we have characterized in the present study. Notably, two rodent models of mild perinatal injury (one preterm mild HI^46^ and one in utero mild hypoxia^47^), which showed no abnormalities on MRI 1–3 months after injury did demonstrate long term behavioral deficits. After our mild injury *in vivo*, it is possible that MRI with advanced sequencing at later timepoints may be more sensitive for any long-term anatomical consequences of mild HI, and subsequent behavioral testing may find correlates to the human deficits after mild perinatal HI. The ‘normal’ cortical activity we measured at 11 days after injury does not predict whether later dysmaturation occurs. Additionally, our *in vivo* field of view for the progression of injury was in the somatosensory cortex rather than the hippocampus, which is the most vulnerable region after moderate to severe HI,^41^ and we may have missed more abundant and different effects on cell death progression and network abnormalities occurring there *in vivo*.

Despite these limitations, our data provide a strong rationale for future studies looking at later timepoints for MRI imaging, neuronal activity and death analyses, and long-term behavioral outcomes. Additionally, we provide an *in vivo* benchmark for how long the therapeutic window is open after mild HI injury and against which therapeutic interventions can be tested to see whether they attenuate ongoing neuronal death. Using the methods developed in this study, one can also determine whether there are functional metrics that predict persistent neuronal death and/or biomarkers of the severity of persistent neuronal death. Since there is likely similar long-term progressive neuronal death occurring in our neonates with mild HIE, acute MRI while the patients are still in the NICU may miss the extent of an injury and not avail them to developing therapeutic interventions. Later repeat neuroimaging is the minimum that can be done to begin to ascertain the actual neurodevelopmental burden of mild HIE. Importantly, this evidence for late, sustained neuronal death following mild HI must be incorporated in decisions regarding possible therapeutic interventions in these patients.

## Supplementary Material

fcaf153_Supplementary_Data

## Data Availability

All processed data associated with this study are present in the paper or in the Supplementary Materials. All raw data is available upon reasonable request. Custom written code generated for the analyses within this manuscript are publicly available online at https://github.com/MGH-Epilepsy-Research-Group.
