# Supplementary material for: Ongoing loss of viable neurons for weeks after mild hypoxia-ischaemia
Source: Brain Commun. 2025 Apr 18;7(2):fcaf153. doi: 10.1093/braincomms/fcaf153 (PMC12034461; doi:10.1093/braincomms/fcaf153)
Supplement: fcaf153_Supplementary_Data [file fcaf153_supplementary_data.pdf]

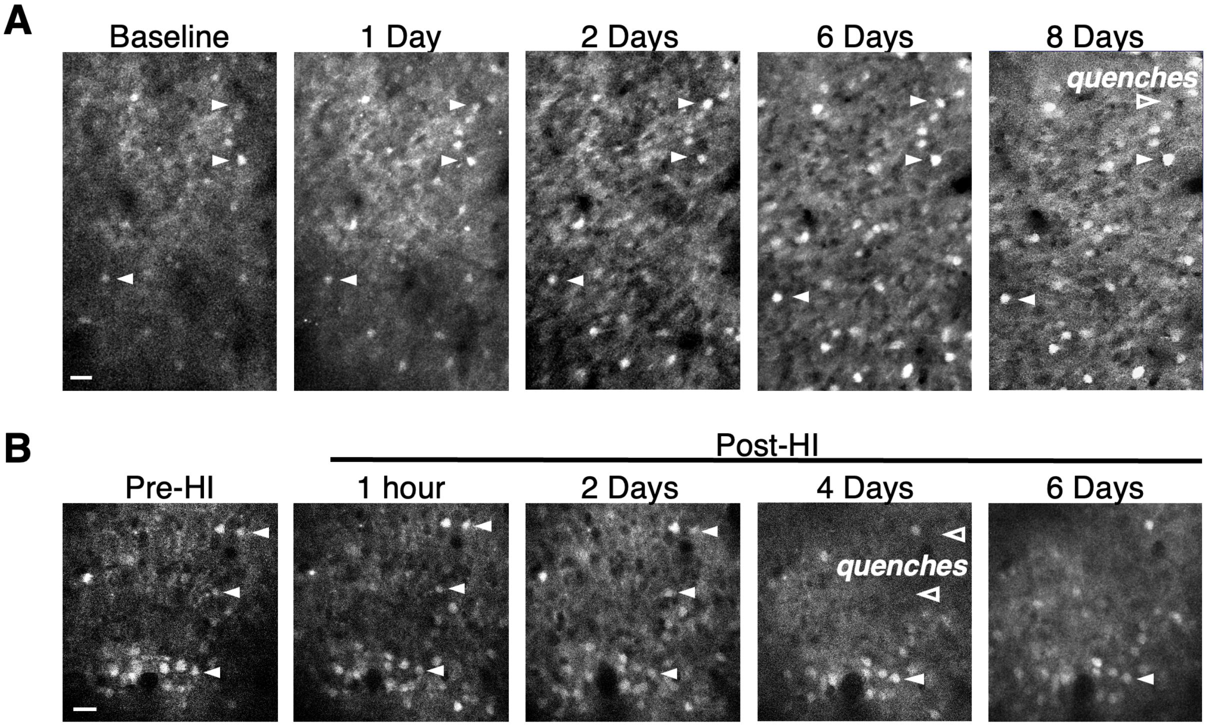

Supplementary Figure 1 Longitudinal tracking of cortical neuronal survival in vivo in a control animal and after hypoxia-ischemia. Unanesthetized, in vivo two-photon (2P) fluorescence images of layer II/III somatosensory cortex over time demonstrating tracking of mRuby-positive cortical neurons from a control (A) and hypoxia-ischemia (HI) (B) animal. White arrowheads show representative tracked neurons. Open white arrowheads show area where tracked neuron has quenched. Scale bars = 25 μm.

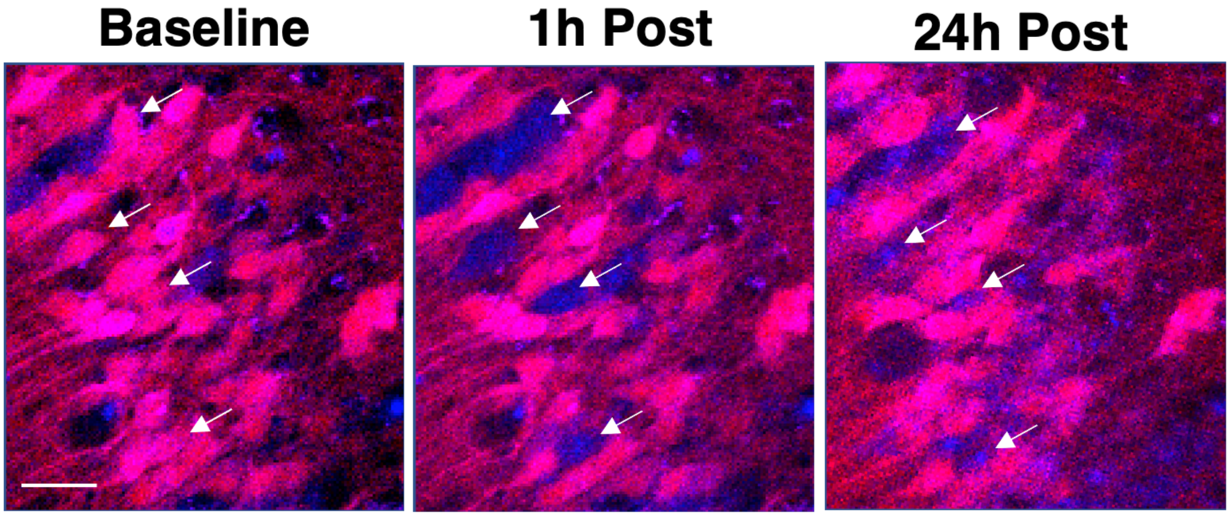

Supplementary Figure 2 Caspase activation in dying hippocampal pyramidal neurons. Representative two-photon (2P) fluorescence images of mRuby-positive hippocampal pyramidal neurons (pink) in vitro incubated with NucView® Blue Caspase-3 dye (blue) in an unhealthy DIV16 organotypic hippocampal slice. Neurons that quenched in unhealthy conditions (arrows) show initial diffuse, cytoplasmic caspase staining and finally nuclear localization showing fragmented nuclear material consistent with apoptotic cell death. Scale bar = 25 μm.

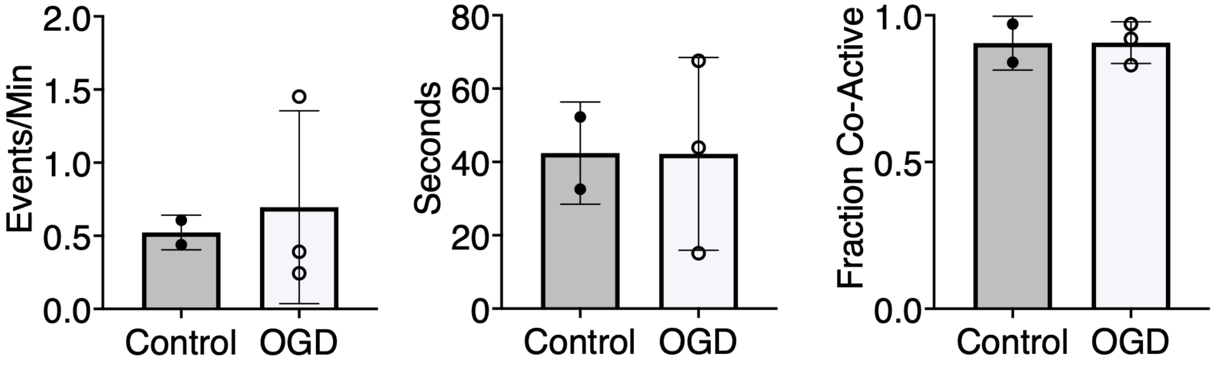

Supplementary Figure 3 In vitro synchronous hippocampal activity was preserved 11-12 days after mild oxygen glucose deprivation. Mean ± standard deviation of network calcium activity metrics in hippocampal slices exposed to oxygen-glucose deprivation (OGD; *n* = 3 hippocampal slices from 3 different pups, open circles) versus control conditions (*n* = 2 hippocampal slices from 2 different pups, filled circles) 11-12 days after injury. The frequency and duration of hippocampal network events per slice is shown. Hippocampal activation represents the average proportion of co-active cells (‘Fraction Co-Active’) per network event per slice. No differences were detected between groups for hippocampal event frequency (t=0.35, df=3, *P* = 0.75, unpaired t-test), hippocampal event duration (t=0.01, df=3, *P* = 0.99, unpaired t-test), and hippocampal activation (t=0.02, df=3, *P* = 0.98, unpaired t-test). Each data point represents the respective values from a single hippocampal slice.

Supplementary Table 1

Animal	Somatosensory Cortex (mm^3)		Ratio (L/R)	Total Volume (mm^3)
	Left	Right		
2.14.24 Sham Pup 1	56	54	0.95	110
2.14.24 Sham Pup 2	57	54	0.95	112
2.14.24 Sham Pup 3	54	53	0.99	106
1.18.24 HI Pup 3	62	64	1.03	126
1.26.24 HI Pup 2	59	57	0.97	117
9.5.23 HI Pup 3	55	52	0.96	107

Animal	Hippocampus (mm^3)		Ratio (L/R)	Total Volume (mm^3)
	Left	Right		
2.14.24 Sham Pup 1	11	11	1.00	22
2.14.24 Sham Pup 2	11	10	1.00	21
2.14.24 Sham Pup 3	10	10	1.00	20
1.18.24 HI Pup 3	11	11	1.00	22
1.26.24 HI Pup 2	10	10	1.00	20
9.5.23 HI Pup 3	10	10	1.00	20

Animal	Cerebral Hemisphere (mm^3)		Ratio (L/R)	Total Volume (mm^3)
	Left	Right		
2.14.24 Sham Pup 1	86	81	0.95	167
2.14.24 Sham Pup 2	86	83	0.97	169
2.14.24 Sham Pup 3	81	79	0.97	160
1.18.24 HI Pup 3	92	94	1.02	187
1.26.24 HI Pup 2	87	86	0.98	173
9.5.23 HI Pup 3	81	79	0.97	161

Supplemental Experimental Procedures

Processing and analysis of calcium imaging

Timelapse recordings were first motion corrected using the Image Stabilizer plugin in Fiji. Epochs where movement was too significant to correct were removed. For network analyses, The Fiji plugin TrackMate[1] was used to automatically select soma ROIs of active, GCAMP+ cells from the frame-to-frame standard deviation (STD) projection, and calcium signal was extracted as the mean pixel intensity of each ROI. For neuronal analyses of manually tracked neurons, the presence and location of individual neurons were confirmed using aligned mRuby images and manual ROIs were drawn in that location on the STD projection of the calcium imaging (thus, able to capture calcium activity of neurons regardless of activity status). Calcium traces were normalized on a frame-by-frame basis as $\Delta F/F$ as : $F_{raw} – F_{min} / F_{min}$. F minimum ($F_{min}$) for each ROI was calculated as the bottom 1st percentile of the mean intensity over all frames. The threshold for being considered in the active state was determined using the kernel density estimation method,[2] with the threshold set at the 90th percentile area under the 1st distribution curve (where the first distribution will largely be comprised of baseline, inactive calcium fluorescence values). To determine what percentage of simultaneously active cells represented a statistically significant network or cortical event, surrogate datasets were constructed by reshuffling activation timing in each cell individually and then averaging the surrogate datasets to create a distribution of event sizes. This was repeated 1000 times and the threshold significance for network or cortical events was then set at $P < 0.01$. The time points where the percentage of cells active exceeded or dropped under the threshold were set to be, respectively, the onset and offset of the network or cortical events. These time points were used to quantify the duration of the events. The maximum percentage of cells simultaneously active during any given network or cortical event was averaged

across all network events in a given recording to measure ‘network/cortical activation’. Using the times of the network events defined by these analyses, the proportion of network events during which individual neurons were active was measured to define ‘neuronal network participation’. Custom written codes for these analyses are publicly available online at https://github.com/MGH-Epilepsy-Research-Group.

Of note, neuropil subtraction was completed on a subset of data and no effect on the conclusions of the results for network, cortical, or individual neuronal analyses were seen. Thus, no neuropil subtraction was applied to the shown analyses. Additionally, no video recording was completed during these experiments and thus the state of the animal was unable to be synchronously verified with the recordings.

Ex vivo magnetic resonance imaging

The scanner was equipped with a 14T magnet with a Bruker Avance Neo Console (Bruker-Biospin, Billerica, MA, 1P BAP117/16). A 60-mm diameter microimaging gradient insert (Resonance Research, Inc., Billerica, MA, BFG-102/60-S12B) was used providing with a peak gradient strength of 1.2T/m. The ex vivo brains were imaged using a Bruker ^{1}H quadrature volume coil (ID=35mm). Multi-slice 2D fast spin echo T2-weighted images were acquired utilizing a Rapid Acquisition with Relaxation Enhancement (RARE) sequence with the following parameters: echo time (TE)/repetition time (TR) = 40/6000 ms, RARE-factor = 1, bandwidth = 50000Hz, in-plane resolution of 50µm x 50µm with 400µm slice thickness, 32 slices, and 8 averages for an acquisition time of 3 hours 12 mins. Field of view was 12mm by 12mm with a 240x240 matrix. During imaging, the field of view was adjusted to ensure similar orientation of

different samples. The slice package was positioned to cover the entire brain and oriented so the ventral line of the brain was parallel to the ventral side of the slice package.

Data was processed using Analysis of Functional Neuroimages (AFNI). Bruker 2dseq images of the RARE images were converted to AFNI format using ‘to3d’. Data was then resampled using “3dresample” to match the resolution of the Allan Brain Atlas. The Allan Brain Atlas was registered to each data to obtain the ROIs using “3dAllineate”. ROIs based on the Allan Brain Atlas were used to calculate the volume of interested areas.

Supplemental References

1. Schindelin J, Arganda-Carreras I, Frise E, et al. Fiji: An open-source platform for biological-image analysis. Nat Methods 2012;9(7):676–682
2. Mitani A, Komiyama T. Real-time processing of two-photon calcium imaging data including lateral motion artifact correction. Front Neuroinform 2018;12; DOI:10.3389/fninf.2018.00098
